# The Immune Responses of the Animal Hosts of West Nile Virus: A Comparison of Insects, Birds, and Mammals

**DOI:** 10.3389/fcimb.2018.00096

**Published:** 2018-04-03

**Authors:** Laura R. H. Ahlers, Alan G. Goodman

**Affiliations:** ^1^School of Molecular Biosciences, Washington State University, Pullman, WA, United States; ^2^Paul G. Allen School for Global Animal Health, College of Veterinary Medicine, Washington State University, Pullman, WA, United States

**Keywords:** innate immunity, West Nile virus, *Culex*, host response, vector-borne disease

## Abstract

Vector-borne diseases, including arboviruses, pose a serious threat to public health worldwide. Arboviruses of the flavivirus genus, such as Zika virus (ZIKV), dengue virus, yellow fever virus (YFV), and West Nile virus (WNV), are transmitted to humans from insect vectors and can cause serious disease. In 2017, over 2,000 reported cases of WNV virus infection occurred in the United States, with two-thirds of cases classified as neuroinvasive. WNV transmission cycles through two different animal populations: birds and mosquitoes. Mammals, particularly humans and horses, can become infected through mosquito bites and represent dead-end hosts of WNV infection. Because WNV can infect diverse species, research on this arbovirus has investigated the host response in mosquitoes, birds, humans, and horses. With the growing geographical range of the WNV mosquito vector and increased human exposure, improved surveillance and treatment of the infection will enhance public health in areas where WNV is endemic. In this review, we survey the bionomics of mosquito species involved in Nearctic WNV transmission. Subsequently, we describe the known immune response pathways that counter WNV infection in insects, birds, and mammals, as well as the mechanisms known to curb viral infection. Moreover, we discuss the bacterium *Wolbachia* and its involvement in reducing flavivirus titer in insects. Finally, we highlight the similarities of the known immune pathways and identify potential targets for future studies aimed at improving antiviral therapeutic and vaccination design.

## Introduction

**Figure 1:**
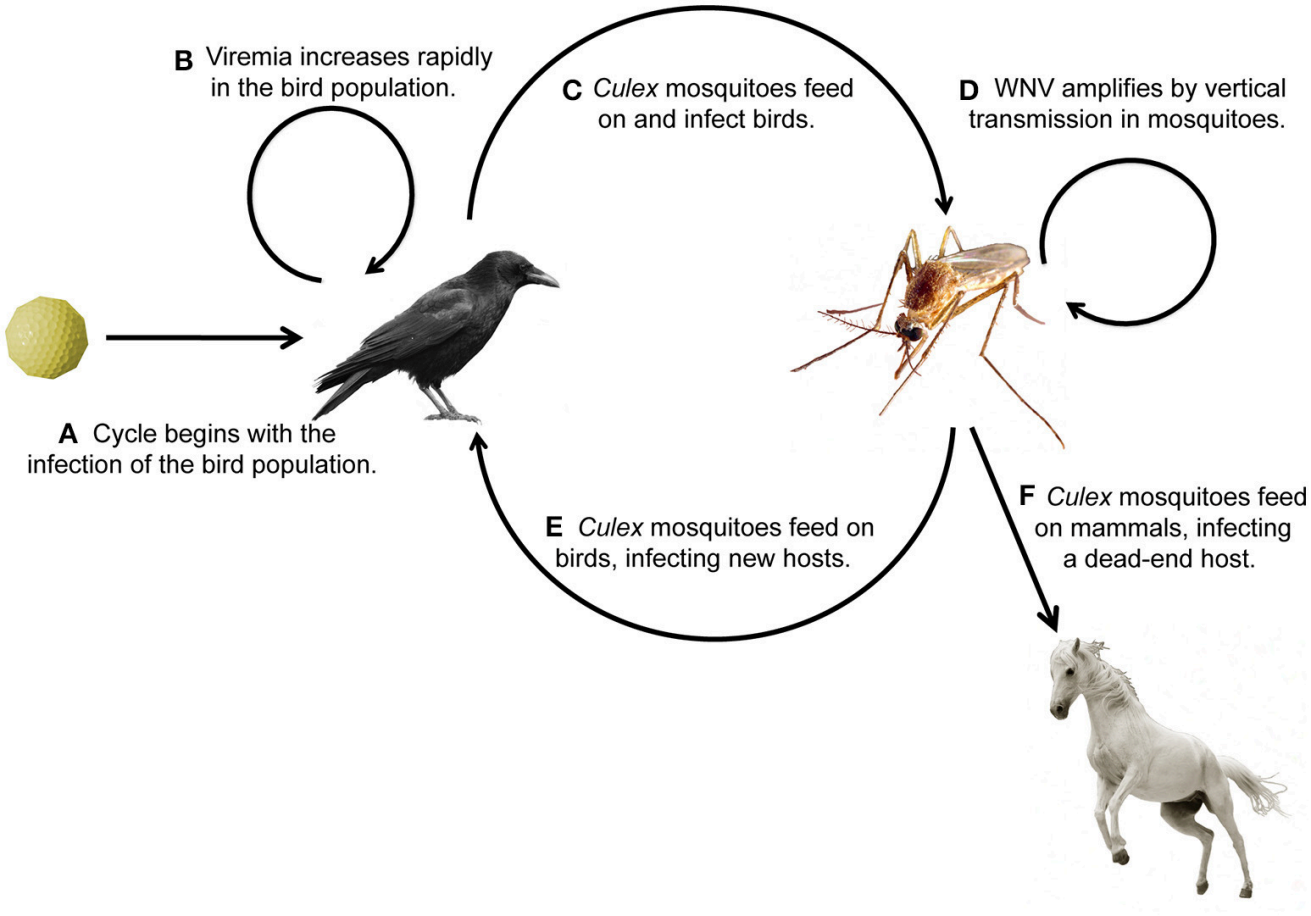
Transmission cycle of West Nile virus through its animal hosts. **(A,B)** Birds become infected with WNV and viral titer increases, **(C,D)** birds transmit the infection to mosquitoes, **(E)** which transmit the infection to birds, causing enzootic infection, or **(F)** bridge the infection to humans and horses, the common dead-end hosts.

West Nile virus (WNV) belongs to the flavivirus genus, which also includes dengue virus (DENV), yellow fever virus (YFV), and Zika virus (ZIKV). WNV is endemic to the United States (U.S.) and Canada, Africa, Europe, the Middle East, and West Asia (WHO, 2011). WNV has a single-stranded positive-sense RNA genome encoding approximately 11,000 nucleotides. It is translated as a polyprotein and processed into 3 structural and 7 nonstructural viral proteins (reviewed in Brinton, 2013). The virus amplifies, or replicates to high titer (Figures 1A,B), within the bird population, making them likely to transmit the infection to mosquitoes (Figure 1C), primarily of the *Culex* genus. Mosquitoes can then reinfect the bird population, further perpetuating enzootic infection (Figure 1E), or can bridge the infection to mammals, most commonly humans and horses (Figure 1F). It is at this interface that public health becomes a concern. Human symptoms can be mild, presenting with headache, weakness, or fever, or more severe, presenting with meningitis or encephalitis (Petersen et al., 2013). In this review, we discuss mosquito populations in North America with particular attention to species that bridge WNV infection to humans, and then survey the innate immune response pathways of the animals commonly infected with WNV: mosquitoes, birds, horses, and humans. While the adaptive immune response is important for mammalian survival to WNV, this review focuses on innate pathways and rapid immune activation during WNV infection. We review possible avenues for therapeutic design, including antibodies for passive immunity and the endosymbiont *Wolbachia* to reduce infection in insects. Lastly, we identify new areas for investigation, especially those focused on vaccine development and disease therapeutics.

## *Culex* mosquitoes as vectors of infection

Mosquitoes in the culicine family carry WNV, and each species has a preferred geographical tropism, blood meal host, and daily and seasonal feeding pattern. *Culex quinquefasciatus* is located between the latitudes 36°N and 36°S (Barr, 1957), and in the U.S. from coast to coast (Darsie and Ward, 2005). *Cx. quinquefasciatus* has been observed as far north as 39°N, giving it some geographical overlap with *Cx. pipiens*, which lives above 36°N (Barr, 1957). *Cx. pipiens* ranges north into British Columbia and through Maine. *Cx. tarsalis* can be found in most of the U.S., but is not usually found in the easternmost states, likely due to competition with *Cx. salinarius*, which prefers warmer, coastal temperatures (Darsie and Ward, 2005). Finally, *Cx. restuans* is found in more urban areas, as the larvae are better able to tolerate pollution than other mosquito species (Johnson et al., 2015). *Culex* population genetics, mating patterns, and host selection, with emphasis on data collected in California, has been reviewed by Reisen (2012), which concludes that urbanization will favor *Cx. pipiens* and hinder *Cx. tarsalis*. While not discussed in Reisen (2012), *Cx. restuans* is also likely to thrive in metropolitan areas, perhaps making *Cx. pipiens* and *Cx. restuans* of greatest importance for the study of vector-borne disease.

Although research on *Culex* mosquitoes in North America has been ongoing for over 70 years, the arrival of WNV to the U.S. spurred deeper research into the species-specific and region-specific differences between *Culex* mosquitoes that allow them to be enzootic vectors (Figure 1E) and bridges to human infection (Figure 1F). *Culex* species feed on avian hosts, either as a primary source of blood meal or more opportunistically (Molaei et al., 2006; Reisen, 2012). A variety of studies have investigated the competence of *Culex* mosquito populations in specific geographic locations to transmit WNV to mammals. In a study completed in Connecticut, researchers found that *Cx. restuans* and *Cx. pipiens* tend to feed on birds, determined by the presence of bird blood meal, making these important species for enzootic infection. Moreover, *Cx. salinarius* is most likely to feed on both vertebrates and birds, making it an important bridge to humans in this region (Molaei et al., 2006). In another study in the northeastern U.S., researchers determined that vector competence can vary over time and is dependent on environmental factors, such as temperature, and genetic factors, such as ancestry. Furthermore, the authors conclude that *Cx. restuans* are more likely to transmit WNV than *Cx. pipiens* (Kilpatrick et al., 2010).

The *Cx. quinquefasciatus* mosquitoes are the primary species in the southern U.S. and Mexico. *Cx. quinquefasciatus* in Cancún and Chetumal often feed on humans, but rarely on birds, so this species is not a likely bridge between bird and human WNV infection (Janssen et al., 2015). However, in East Baton Rouge Parish, Louisiana, *Cx. quinquefasciatus* frequently feed on avian hosts, as well as human and other mammalian sources of blood meal, suggesting that *Cx. quinquefasicatus* is a vector for human transmission of WNV in southern Louisiana (Mackay et al., 2010). In Bernalillo County, New Mexico, researchers determined that *Cx. quinquefasciatus* mosquitoes are likely the primary vectors for enzootic infection in birds, but *Cx. tarsalis* are more likely the bridge to infect humans, as these mosquitoes feed on both mammals and birds throughout the feeding season (Lujan et al., 2014).

Laboratory studies have further investigated the species-specific differences between *Culex* mosquitoes when infected with WNV. *Cx. tarsalis* infected with WNV have decreased fecundity and increased feeding rates, but no change in survival (Styer et al., 2007). Conversely, *Cx. pipiens* show no difference in survival, fecundity, or feeding rates when infected with WNV (Ciota et al., 2011). These findings complicate the ability to predict WNV infection rates in specific mosquitoes. Ciota et al. predicts that susceptible *Cx. pipiens* mosquitoes will be maintained in a community, as there is no cost for infection, but there is a cost for resistance. The results in Styer et al. suggest that in *Cx. tarsalis* the cost for infection, namely decreased fecundity, may be overcome by an increase in feeding rate. This behavioral compensation is supported by the finding that *Cx. tarsalis* have a higher estimated rate of transmission than *Cx. pipiens* (Turell et al., 2001, 2002). The same research group determined that *Cx. restuans* and *Cx. salinarius* are both efficient vectors of WNV infection, while *Cx. quinquefasciatus* is moderately efficient (Sardelis et al., 2001). Additionally of note, *Cx. pipiens* can perpetuate WNV infection by vertical transmission (Dohm et al., 2002); consequently multiple generations of mosquitoes are infective (Figure 1D).

Alarmingly, the geographic range of *Culex* mosquitoes is expanding. Models of *Cx. pipiens* and *Cx. tarsalis* reveal that climate change is likely to contribute to the expansion of the mosquito population in Canada and extend the WNV transmission season by the year 2050 (Hongoh et al., 2012; Chen et al., 2013). Additionally, *Cx. quinquefasciatus* season is predicted to increase in length by a few weeks at both the beginning and end of the summer in the U.S. (Morin and Comrie, 2013). Taken together, these models conclude that the geographic range of mosquitoes, and consequently WNV infections, will increase. A summary of the effects of climate change on several insect-borne infections is provided in Andersen and Davis (2017).

## The mosquito immune response to WNV

Mosquitoes utilize an innate immune response to WNV to prevent mortality from infection. The RNA interference (RNAi) pathway is conserved across diverse phyla and provides host protection against virus infection, including arboviruses (Olson and Blair, 2015). Dicer-2, the viral nucleic acid sensor of the RNAi pathway, is utilized in the response to WNV infection in *Cx. quinquefasciatus* cells, and orally-infected *Cx. quinquefasciatus* mosquitoes respond to WNV (Kunjin strain) challenge via the RNAi pathway (Paradkar et al., 2014). In fact, WNV (Kunjin strain) has been shown to antagonize the host RNAi response in *Cx. quinquefasciatus* by generating viral noncoding sfRNA (subgenomic flavivirus RNA) that interacts with Dicer and Argonaute 2 (Moon et al., 2015). sfRNA is viral genomic RNA that resists degradation by the host cell by forming pseudoknot structures (Jones et al., 2012; Chapman et al., 2014). The RNAi pathway even drives WNV population diversity in both mosquitoes and *Drosophila melanogaster*, as the RNAi pathway selects for the more diverse virus variant (Brackney et al., 2009, 2015). Like mosquitoes, *D. melanogaster* utilize the RNAi pathway for resistance to WNV infection, determined by the detection of siRNA (small interfering RNA) (Chotkowski et al., 2008), validating the fruit fly as a possible model organism to study mosquito immunity.

Mosquitoes also utilize the JAK/STAT pathway in the immune response to WNV. Transcriptional profiling reveals that *Aedes aegypti* mosquitoes utilize this pathway in response to WNV, DENV, and YFV (Colpitts et al., 2011). Mechanistically, in *Culex* cells the immune response to WNV utilizes a secreted molecule called Vago that, like interferon in mammals, is hypothesized to act as a second messenger to activate the JAK/STAT pathway (Paradkar et al., 2012). Finally, apoptosis, a conserved immediate immune response, occurs in the salivary glands and midgut of *Cx. quinquefasciatus* mosquitoes to control viral load (Vaidyanathan and Scott, 2006; Girard et al., 2007).

Lastly, of note, the endosymbiont *Wolbachia* affects the mosquito host response to WNV. *Wolbachia* is a bacterium originally identified in *Cx. pipiens* (Hertig and Wolbach, 1924), reviewed in Johnson (2015). It is estimated that 40% of all arthropod species (Zug and Hammerstein, 2012) and 7% of *Cx. pipiens* mosquitoes in California (Rasgon and Scott, 2004) are infected with *Wolbachia*. There are a few strains of *Wolbachia* used in laboratory experiments, discussed in Woolfit et al. (2013): *w*Mel was identified in *D. melanogaster* and is benign (Teixeira et al., 2008), *w*MelPop was identified in *D. melanogaster* and has a pathogenic effect (Min and Benzer, 1997), and *w*MelPop-CLA is a strain of *w*MelPop adapted for *Ae. aegypti* (McMeniman et al., 2008). Inaugural experiments in *D. melanogaster* determined that *Wolbachia* infection increases host resistance to the Drosophila C virus, Nora virus, Flock House virus, and WNV (Teixeira et al., 2008; Glaser and Meola, 2010). Subsequently, others determined that the same effect occurs in mosquitoes: The presence of *Wolbachia* (*w*Mel and *w*MelPop-CLA strains) in *Ae. aegypti* mosquitoes is correlated with a reduction in DENV titer (Walker et al., 2011) and WNV titer (Hussain et al., 2013). One study determined that the amount of secreted WNV decreases significantly in *Aedes* cells that are also infected with *Wolbachia*, indicating restriction of the virus. Furthermore, it determined that the *Wolbachia* strain *w*MelPop, but not *w*Mel, has an inhibitory effect on WNV infection *in vivo* (Hussain et al., 2013). Taken together, the strain of *Wolbachia* is important for inhibition of WNV in *Aedes* mosquitoes.

Perhaps of greater biological importance for WNV is the effect of *Wolbachia* on *Culex* mosquitoes. In a study using *Cx. quinquefasciatus*, researchers concluded that *Wolbachia* increases host resistance to WNV infection (Glaser and Meola, 2010). However, this is in contrast to another study that suggests that the presence of *Wolbachia* (*w*AlbB) can increase WNV titer in *Cx. tarsalis* (Dodson et al., 2014). *w*AlbB is a strain originally isolated from *Ae. albopictus* (Zhou et al., 1998).

Because of the controversial results, one study specifically compared the effects of *Wolbachia* strain *w*AlbB in *Ae. aegypti* on DENV and WNV (Kunjin strain). The study concluded that both somatic infection and stable transinfection of *Wolbachia* lead to inhibition of DENV and WNV replication and transmission (Joubert and O'Neill, 2017). Some researchers even suggest that introducing *Wolbachia* into the wild mosquito population will reduce DENV infection in humans (Schmidt et al., 2017), and modeling predicts that WNV could be eradicated subsequent to the introduction of *Wolbachia* in the ecosystem (Farkas et al., 2017).

Because *Wolbachia* is a bacterium, it would follow that it is priming an immune response in mosquitoes. However, this does not seem to be the case during DENV infection (Rancès et al., 2012). Rainey et al. describe hypotheses for the mechanism by which *Wolbachia* reduces viral titer (Rainey et al., 2014). One possible mechanism of antiviral action could be competition between *Wolbachia* and a virus for cellular resources. This is supported by Moreira et al. (2009) which determined that *Wolbachia* and DENV are not found in the same cells. Another putative mechanism is modulation of the autophagy pathway. DENV utilizes the autophagy pathway for replication (Lee et al., 2008), however, *Wolbachia* (*w*AlbB) has been shown to manipulate this pathway for its own survival (Voronin et al., 2012). This mechanism may not be relevant for all flaviviruses, as WNV does not utilize autophagy for replication in mammalian cells (Vandergaast and Fredericksen, 2012). More work will need to be completed in the insect model to determine the role of autophagy in WNV pathogenesis.

## The bird immune response to WNV

Birds are an important reservoir of WNV, as the virus replicates to high titers in several bird species (Figure 1B; Komar et al., 2003). Additionally, the migration of bird populations aids in the distribution of WNV beyond the range of mosquitoes (Reed et al., 2003; Owen et al., 2006). Similarly to human and horse immunity, birds utilize the 2′-5′-oligoadenylate synthase (OAS) pathway in the immune response to WNV. Briefly, OAS proteins detect double-stranded RNA from viruses and undergo a conformational change to synthesize 2′-5′-oligoadenylates. These second messengers bind inactive RNase L, which then dimerizes to become active and cleave viral RNA. The OAS response is often utilized during flavivirus and alphavirus infections, likely because these positive-sense RNA viruses develop double-stranded RNA as replication intermediates in higher concentrations, as compared to a negative-sense RNA virus (Silverman, 2007). This response pathway ultimately inhibits the virus and induces apoptosis (Castelli et al., 1998; Tag-El-Din-Hassan et al., 2012).

While antibodies are a hallmark of adaptive immunity, passive immunity is a form of rapid immune activation, similar to innate immunity. Several bird species develop neutralizing antibodies to WNV, with long-lasting protection over multiple WNV seasons, including the house sparrow (*Passer domesticus*) (Nemeth et al., 2009) and raptor species (Nemeth et al., 2008). Importantly, young chicks can receive maternally-inherited passive immunity for rapid protection from virus infection. Maternally-inherited antibodies to WNV have been measured in flamingo chicks (*Phoenicopterus chilensis* and *Phoenicopterus ruber ruber*) (Baitchman et al., 2007), Eastern screech owls (*Megascops asio*) (Hahn et al., 2006), and rock pigeons (*Columba livia*; Gibbs et al., 2005), indicating that this is an effective strategy for protecting chicks.

Additionally, there is some cross-protection in birds to multiple flavivirus types. House finches that are first challenged with St. Louis encephalitis virus (SLEV) first and then WNV have an antibody response to WNV. Interestingly, finches first challenged with WNV and then SLEV have an elevation in WNV antibody titers, but no increase in SLEV antibody titers during the second infection (Fang and Reisen, 2006). This information could be useful in vaccine design to protect birds against flavivirus infection. Perhaps antibodies to WNV could confer resistance to multiple flaviviruses, theorizing a universal flavivirus vaccine. Some researchers did vaccinate birds with the goal of saving rare species. One study used a DNA vaccine to protect captive California condors (*Gymnogyps californianus*) during the initial spread of WNV in the U.S. The study determined that the vaccine is safe for California condors, stimulates protective antibodies, and protects against naturally circulating WNV (Chang et al., 2007).

## The horse immune response to WNV

Horses are also susceptible to WNV infection (Figure 1F), and since its entry to the U.S. in 1999, WNV has caused 27,726 confirmed equine cases (data through 2016; USDA APHIS, 2017). In a WNV outbreak in 2002, a survey determined that 22% of infected horses died from infection (Schuler et al., 2004). Like humans, horses have a robust immune response to WNV that utilizes both the innate and adaptive responses.

In the early immune response, horses utilize an interferon-mediated (IFN) response. In one study that used WNV (Kunjin subtype) authors found increased levels of type I and type II interferon in blood leukocytes, lymph nodes, and spleen. They also noted increases in IFN-α, CXCL10, TLR3, ISG15, and IRF-7 in the brain, but no neuroinvasion of the virus (Bielefeldt-Ohmann et al., 2017). In a project that investigated global gene expression of the central nervous system (CNS) of horses by sequencing the transcriptome of the brain and spinal cord, researchers identified gene ontology groups utilized in the WNV immune response. These include IL-15, IL-22, MAPK, and JAK/STAT signaling, as well as apoptosis pathways and B cell and T cell receptor expression (Bourgeois et al., 2011). These pathways also exist in humans, indicating similarities between the human and horse immune responses to WNV.

Horses also have an OAS1 response to WNV that is inducible by interferon, and variation in the horse *OAS1* gene has been associated with changes in WNV susceptibility (Rios et al., 2007, 2010). Furthermore, like birds, horses also mount an immune response to WNV using antibodies (Bielefeldt-Ohmann et al., 2017). Pony foals have been shown to receive maternally-inherited antibodies as a means of passive immunity (Wilkins et al., 2006). This strategy utilizes antibodies to rapidly activate the immune response to protect foals from infection.

## The human immune response to WNV

Because of the negative impact of WNV on the human population throughout the U.S., many researchers have characterized the human immune response to WNV, reviewed by Suthar et al. (2013). Briefly, the viral RNA sensors RIG-I and MDA5 detect WNV intracellularly, activating the adaptor protein MAVS, leading to IRF-3 activation for interferon induction and downstream induction of interferon-stimulated genes (ISGs) (Fredericksen et al., 2004, 2008; Fredericksen and Gale, 2006). The cytokines IFN-α and IFN-β are important for controlling WNV tropism by inducing an antiviral state (Samuel and Diamond, 2005). Priming an IFN response with the unrelated virus Invertebrate Iridescent virus 6 actually reduces WNV (Kunjin strain) titer *in vitro* (Ahlers et al., 2016). The downstream ISGs include the IFIT (interferon-induced protein with tetratricopeptide repeats) genes and viperin, which inhibit viral infection and replication (Jiang et al., 2010; Szretter et al., 2011; Gorman et al., 2016). Notably, the nonstructural protein NS5 of WNV inhibits the interferon response by preventing the expression of IFN-α receptor 1 on the surface of host cells (Lubick et al., 2015). The virus can also evade host restriction by IFIT proteins via 2'-O methylation of WNV (Daffis et al., 2010; Szretter et al., 2012). This strategy of antagonizing the IFN response is common to flaviviruses. ZIKV and DENV NS5 target human STAT2 for degradation (Morrison et al., 2013; Grant et al., 2016) by different mechanisms. YFV binds to STAT2 after host cells are stimulated with IFN to prevent it from binding to promoter elements (Laurent-Rolle et al., 2014).

Apoptosis is another innate immune response in mammals that restricts WNV replication, and the mechanisms of apoptosis induction have been studied in murine models. In one, mouse embryonic fibroblasts utilize CHOP (cyclic AMP response element-binding transcription factor homologous protein) to induce apoptosis and reduce WNV titer (Medigeshi et al., 2007). However, while apoptosis can be an effective method for eliminating virus from a host, it has a damaging effect on neurons. Although caspase 3 is activated during WNV infection, possibly in an attempt at an immune response, caspase 3 knockout mice have higher survival during WNV infection and less neuronal death than their wild-type counterparts (Samuel et al., 2007). Moreover, inhibition of caspase 8 during WNV infection reduces CNS tissue injury (Clarke et al., 2014). These findings suggest that the net beneficial or detrimental outcome of apoptosis as an immune response could be dependent on the type of tissue and the specific pro-apoptotic pathway activated.

While the interferon response is critical for restricting WNV, the human immune response to WNV also utilizes the OAS and RNase L pathway (Hornung et al., 2014). Indeed, a single nucleotide polymorphism in the *OAS1b* gene, namely rs34137742, that contains a C to T substitution in the second intron of the gene, is a risk factor for human West Nile encephalitis and paralysis from WNV infection (Bigham et al., 2011). *OAS1* has been demonstrated to undergo positive selection in Old World primates (Fish and Boissinot, 2016), indicating a historic interaction between flaviviruses like WNV and host immunity (Daugherty and Malik, 2012). This pathway is conserved in birds and horses, as discussed in earlier sections.

Passive immunity is also useful for rapid host protection to WNV. B cell and antibody-deficient (μMT) mice and B cell activating factor receptor (BAFFR)-deficient mice are susceptible to infection, but, if treated with immune sera from a wild-type mouse with antibodies to WNV, can be protected from infection (Diamond et al., 2003; Giordano et al., 2017). Strikingly, the BAFFR-deficient mice can develop sustained protective immunity after treatment with immune sera (Giordano et al., 2017). Together, this indicates that passive immunity could be utilized as a therapeutic option for human infection to induce a robust immune response. To the best of our knowledge, no studies have determined if antibodies to WNV are maternally-inherited in humans.

In summary, the animal hosts of WNV have both shared and divergent immune response pathways (Table 1). While mammals do possess an RNAi pathway like insects, the IFN immune response takes precedence as the primary innate immune response (Benitez et al., 2015). Both insects and mammals utilize apoptosis as a rapid response to virus infection. Birds, horses, and humans all utilize an OAS response and passive immunity, which are both activated rapidly during infection and are effective at restricting WNV.

**Table 1:** Summary of the host responses of the animal hosts of West Nile virus.

	**Mosquito**	**Bird**	**Horse**	**Human**
RNAi response	Yes	Unknown	Unknown	Not utilized
Interferon-mediated response	Possibly, using Vago	Unknown	Yes	Yes
Apoptosis	Yes	Unknown	Yes	Yes
OAS response	Absent	Yes	Yes	Yes
Passive immunity	Absent	Yes	Yes	Yes

*Various classes of conserved host responses are noted if they are utilized in the response to WNV (yes), present in the host, but not utilized in the response to WNV (not utilized), or not present/undiscovered in the host (absent)*.

## The future of WNV research

Presently, no approved vaccine or therapeutic exists for human use to prevent or treat WNV infection. There are, however, four approved horse vaccines in use in the U.S., which have greatly aided in the reduction of equine cases. Veterinary options include two inactivated whole virus vaccines, a non-replicating live recombinant canary pox vector vaccine, and an inactivated flavivirus chimera vaccine (Ishikawa et al., 2014; Balasuriya et al., 2015). A number of human vaccines have been proposed, with some in clinical trials.

One promising vaccine is ChimeriVax-WN02, which is a live, attenuated vaccine created by inserting the genes for the pre-membrane (prM) and envelope (E) proteins from WNV into the yellow fever 17D clone (Arroyo et al., 2004). The vaccine completed a successful phase I clinical trial and two phase II clinical trials (Monath et al., 2006; Biedenbender et al., 2011; Dayan et al., 2012). Another chimeric vaccine that passed a phase I trial, rWN/DEN4Δ30, also utilizes prM and E from WNV but uses the live attenuated vaccine candidate rDEN4 Δ30 as a vector (Durbin et al., 2013). Another strategy utilizes a DNA vaccine with the prM and E proteins of WNV, either with the CMV promoter (Martin et al., 2007) or a modified CMV promoter (CMV/R) (Ledgerwood et al., 2011). Both versions of this vaccine completed successful phase I clinical trials.

Despite these successful early clinical trials, no WNV vaccine has moved into phase III trials. Some challenges for a phase III trial for a WNV vaccine are discussed in Ishikawa et al. (2014). One notable impediment is the low and sporadic incidence of WNV activity, which would make it difficult to establish vaccine efficacy. Because of the logistical challenges of developing and licensing a vaccine for WNV, perhaps a more feasible avenue for prevention is the introduction of *Wolbachia* into the mosquito population. As discussed in an earlier section, *Wolbachia* reduces flavivirus titer in mosquitoes, and models predict that WNV eradication is possible with the introduction of *Wolbachia* (Farkas et al., 2017). Certainly, great caution should be taken to determine if the introduction of *Wolbachia* into the *Culex* population would have any detrimental effects on the greater ecosystem.

## Author contributions

LA wrote the manuscript in consultation with AG.

### Conflict of interest statement

The authors declare that the research was conducted in the absence of any commercial or financial relationships that could be construed as a potential conflict of interest.
